# Primary Retroperitoneal Lymph Node Dissection for Clinical Stage II A/B Seminomas: A Systematic Review and Meta-Analysis

**DOI:** 10.1590/S1677-5538.IBJU.2024.0134

**Published:** 2024-04-25

**Authors:** Bárbara Vieira Lima Aguiar Melão, Lucas Guimarães Campos Roriz de Amorim, Murilo Ribeiro Sanches, Giovanna Veiga Gomes, Douglas Mesadri Gewehr, Luis Henrique de Oliveira Moreira, Thaise Pedreira da Silva, Matheus de Melo Lobo, Gustavo Ruschi Bechara

**Affiliations:** 1 Divisão de Urologia Universidade de São Paulo São Paulo SP Brasil Divisão de Urologia, Universidade de São Paulo, São Paulo, SP, Brasil;; 2 Divisão de Urologia Universidade Federal de Minas Gerais Belo Horizonte MG Brasil Divisão de Urologia, Universidade Federal de Minas Gerais, Belo Horizonte, MG, Brasil;; 3 Departamento de Medicina Universidade Federal de Goiás Goiânia GO Brasil Departamento de Medicina, Universidade Federal de Goiás, Goiânia, GO, Brasil;; 4 Departamento de Medicina Universidade Municipal de São Caetano do Sul São Caetano do Sul SP Brasil Departamento de Medicina, Universidade Municipal de São Caetano do Sul, São Caetano do Sul, SP, Brasil;; 5 Instituto do Coração de Curitiba Curitiba PR Brasil Instituto do Coração de Curitiba, Curitiba, PR, Brasil;; 6 Departamento de Medicina Universidade Federal de Minas Gerais Belo Horizonte MG Brasil Departamento de Medicina, Universidade Federal de Minas Gerais, Belo Horizonte, MG, Brasil;; 7 Departamento de Cirurgia Hospital Santa Izabel Salvador Brasil Departamento de Cirurgia, Hospital Santa Izabel, Salvador, Brasil;; 8 Divisão de Cirurgia Oncológica A.C. Camargo Cancer Center São Paulo SP Brasil Divisão de Cirurgia Oncológica, A.C. Camargo Cancer Center, São Paulo, SP, Brasil;; 9 Divisão de Urologia Universidade Federal do Espírito Santo Vitória ES Brasil Divisão de Urologia, Universidade Federal do Espírito Santo, Vitória, ES, Brasil

**Keywords:** Testicular Neoplasms, Seminoma, Male Germ Cell Tumor [Supplementary Concept]

## Abstract

**Introduction:**

Chemotherapy and radiation therapy are considered standard treatments for stage II seminoma patients; however, these therapies are associated with long-term toxicities. Recently, retroperitoneal lymph node dissection has emerged as an alternative strategy, and the first three phase II trials were published in 2023 with promising results. The present study conducted a systematic review and meta-analysis to evaluate this surgery as an alternative treatment for stage IIA/B seminoma patients.

**Purpose:**

Seminomas are the most common testicular tumors, often affecting young adult males. Standard treatments for stage II seminomas include chemotherapy and radiation therapy, but these therapies are associated with long-term toxicities. Thus, identifying alternative strategies is paramount. Herein, we conducted a systematic review and meta-analysis to appraise the efficacy and safety of retroperitoneal lymph node dissection (RPLND) for treating this condition.

**Methods:**

We systematically searched the PubMed, Embase, and Cochrane databases for studies evaluating RPLND as a primary treatment for stage II A/B seminomas. Using a random-effects model, single proportion and means and pooled 2-year recurrence-free survival rates with hazard rates and 95% CI were calculated.

**Results:**

Seven studies were included, comprising 331 males with stage II seminomas. In the pooled analysis, the recurrence rate was 17.69% (95% CI 12.31–24.75), and the 2-year RFS rate was 81% (95% CI 0.77–0.86). The complication rate was 9.16% (95% CI 6.16–13.42), the Clavien–Dindo > 2 complication rate was 8.83% (95% CI 5.76–13.31), and the retrograde ejaculation rate was 7.01% (95% CI 3.54–13.40). The median operative time was 174.68 min (95% CI 122.17–249.76 min), median blood loss was 105.91 mL (95% CI 46.89–239.22 mL), and patients with no evidence of lymph node involvement ranged from 0–16%.

**Conclusions:**

Primary RPLNDs for treating stage IIA/B seminomas have favorable RFS rates, with low complication and recurrence rates. These findings provide evidence that this surgery is a viable alternative therapy for these patients.

## INTRODUCTION

Testicular cancer accounts for approximately 1% of malignancies in males, emerging as the predominant solid tumor between the second and fourth decades of life, with an increasing incidence in the later years ( 1 , 2 ). Most men with testicular cancer present a low-stage disease at the time of diagnosis [limited to the testis and retroperitoneum, clinical stages (CSs) I-IIB]. Seminomas are the most common testicular germ cell tumors (GCTs), comprising 50% of all testicular cancers. This disease predominantly affects young adult males, with the highest incidence in the fourth decade ( 3 , 4 ). Current guidelines recommend chemotherapy or radiation therapy as standard therapeutic modalities for CS IIA/B seminomas, defined by enlarged retroperitoneal lymph nodes of up to 5 cm ( 5 , 6 ). These treatments are effective, with a disease-free survival rate of up to 90%. However, these therapies are associated with long-term toxicities, diminished quality of life, and potential secondary tumor development ( 7 - 10 ).

Recently, retroperitoneal lymph node dissection (RPLND), which is well-defined as the primary treatment for non-seminomatous testicular cancer, has emerged as an alternative strategy in CS IIA/B seminomas ( 5 , 6 ). Despite promising results observed with RPLND in these cases, the current evidence remains insufficient to make definitive recommendations regarding its suitability and effectiveness as a treatment option. Therefore, we performed a systematic review and meta-analysis to evaluate RPLND as an alternative option for patients with CS IIA/B seminomas.

## MATERIALS AND METHODS

A systematic review and meta-analysis were performed and reported following the Cochrane Collaboration Handbook for Systematic Reviews of Interventions and the Preferred Reporting Items for Systematic Reviews and Meta-Analysis (PRISMA) Statement guidelines ( 11 , 12 ). The prospective protocol was registered in the International Prospective Register of Systematic Reviews (PROSPERO; ID CRD42023483103).

### Data source and search strategy

We systematically searched PubMed (MEDLINE), Embase, and the Cochrane Central Register of Controlled Trials from inception to February 04, 2024. The search terms included “seminoma” and “retroperitoneal lymphadenectomy”. After removing duplicates, two authors (B.V.L.A.M. and M.R.S.) screened the titles and abstracts and independently assessed full-text articles for inclusion based on prespecified criteria. Discrepancies were resolved in a discussion panel with the senior author. Moreover, we utilized a snowballing technique to search for additional eligible studies by reviewing the references from articles identified in the original search.

### Eligibility criteria

We considered studies eligible for inclusion if they were prospective or retrospective, enrolled patients diagnosed with CS IIA/B testicular seminoma, evaluated primary RPLND, and presented data regarding any of the prespecified endpoints of interest. Exclusion criteria included no outcomes of interest, CS I, IIC, or higher seminomas, and/or failing to specify the CS of the seminomas. Additionally, cohorts or case series with fewer than ten patients were excluded from the pooled analysis due to the high possibility of selection bias ( 13 ).

### Data extraction

Two authors (B.V.L.A.M. and G.V.G.) independently extracted the data for each study using a standardized document to collect the following characteristics: inclusion and exclusion criteria, follow-up period, baseline characteristics, CS, surgical approach, dissection templates, pathologic nodal stage, upstaging, endpoint data, and endpoint definitions. Baseline characteristics were reported as the median and interquartile range for continuous variables and proportion for binary variables.

### Endpoints

Our prespecified primary endpoints were the 2-year recurrence-free survival (RFS) rate, the recurrence rate, and the complication rate according to the Clavien–Dindo (CD) classification ( 14 ). Our secondary outcomes included the retrograde ejaculation rate, complications CD grade > 2, operative time (minutes), estimated blood loss (mL), and length of hospital stay (days).

### Quality assessment

We used the Cochrane Collaboration tool Risk Of Bias In Non-randomized Studies - of Interventions (ROBINS-I) for the quality assessment of individual studies, according to the recommendations from the Cochrane Handbook for Systematic Reviews of Interventions ( 11 , 15 ). Each trial underwent a risk of bias score evaluation, indicating whether there was a serious, moderate, low, or unclear risk of bias across five domains: confounding, selection of participants, classification of interventions, deviations from intended interventions, missing data, measurement of outcomes, and selection of reported results. The two authors independently conducted the assessments (M.M.L. and B.V.L.A.M.), and consensus resolved disagreements.

### Statistical analysis

We summarized single proportions ( *metaprop* ) and single means ( *metamean* ) using an inverse-variance random-effects model, reporting overall proportion and mean with a 95% confidence interval (CI) as a measure of effect size ( 16 ). The exact or Clopper-Pearson method was used to establish 95% CIs for proportion from the selected individual studies ( 17 ). We applied the logit transformation (“PLOGIT”) and log transformation (“MLN”) to normalize the results before calculating the pooled proportion and means, respectively ( 16 , 18 ). In cases of continuous endpoints, which are reported only as medians (interquartile ranges), we estimated corresponding means ± standard deviations by applying the Wan and Luo method ( 19 , 20 ). Furthermore, we pooled the 2-year RFS rates (metagen) using an inverse-variance random-effects model with hazard rates (i.e., RFS) and a 95% CI ( 16 ). The restricted maximum likelihood estimator was used to calculate heterogeneity variance τ^2^.

The RFS rates and 95% CIs from the published Kaplan–Meier (KM) curves were estimated using the highly accurate method of Liu et al. along with the Shiny application to extract raw data coordinates and reconstruct the individual patient data (IPD) from published KM curves ( 21 ). We also performed a leave-one-out sensitivity analysis for the 2-year RFS rate by iteratively omitting one study at a time to ensure the results were not dependent on a single study. We used R version 4.2.3 (The R Foundation for Statistical Computing, MO, USA) and the extension package “meta” for all calculations and graphics ( 16 ).

## RESULTS

### Study selection and characteristics

**Figure 1:**
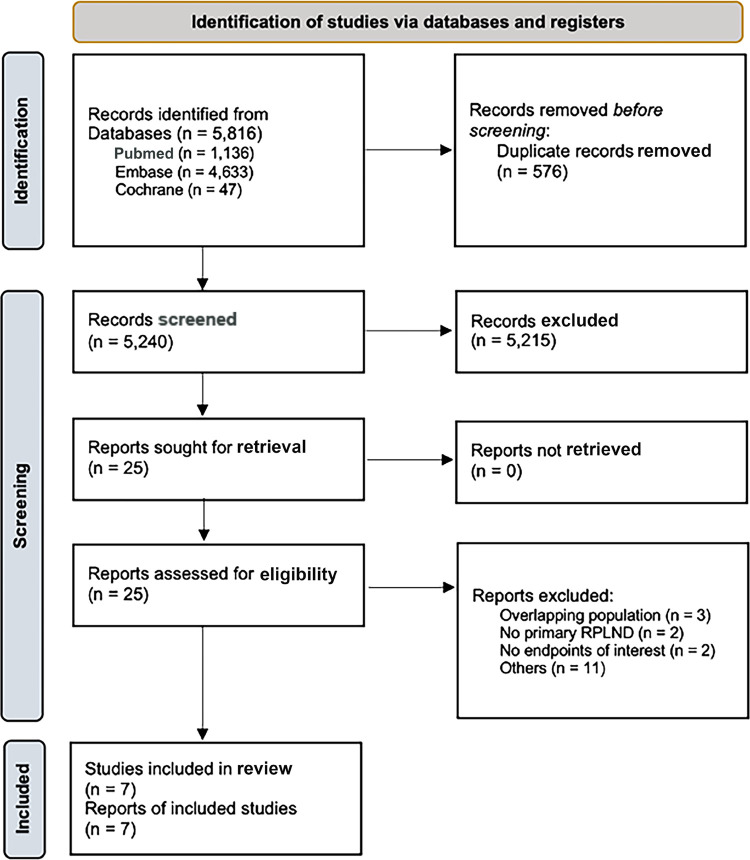
- PRISMA flow diagram of study screening and selection.

Our initial search yielded 5,816 potential articles ( Figure-1 ). After removing duplicates, twenty-five articles were retrieved and reviewed in full for possible inclusion. Seven studies were ultimately included in the pooled analysis (22–28). Four studies were prospective trials (three phase 2 clinical trials), and three were retrospective. The main characteristics of the included studies are presented in Table-1 .

RPLND, retroperitoneal lymph node dissection.

**Table 1:** Main characteristics of the included studies.

Study	Design	RP adenopathy	Tumor markers	Exclusion criteria	Templates of dissection	Adjuvant therapy
**Daneshman et al. ( 22 ), 2023 (SEMS)**	Prospective, single-arm, multicenter, phase 2 clinical trial	1-3 cm, maximum 2 lymph nodes, ipsilateral, synchronous or metachronous*	AFP, ß-hCG, LDH elevation ? 1.5x ULN	Second primary tumor, previous CT/RT, comorbidities precluding surgery	Right: lateral limits from right to left ureter above mesenteric artery and from right ureter to aorta below mesenteric artery Left: from left ureter to inferior vena cava above inferior mesenteric artery and from left ureter to aorta below inferior mesenteric artery	None✝
**Heidenreich et al. ( 23 ), 2024 (COTRIMS)**	Prospective, single-arm, single-center, phase 2 clinical trial	1-5 cm, ipsilateral, synchronous or metachronous	AFP < 5.8 kU/L ß-hCG < 5 U/L LDH < 1.5x ULN	Previous CT	Right: precaval, paracaval, retrocaval, interaortocaval and lateral to the common iliac vessels (crossing of the ureter as caudal boundary, the ureter as lateral boundary and renal vein as a cranial boundary) Left: preaortic area up to the inferior mesenteric artery, para-aortic, and retroaortic areas (crossing of the ureter with iliac artery as the caudal and the lateral boundaries)	None
**Hiester et al. ( 24 ), 2023 (PRIMETEST)**	Prospective, single-arm, single-center phase 2 clinical trial	< 5 cm, ipsilateral, synchronous or metachronous	Normal AFP ß-hCG < 5 U/L	Previous scrotal or RP surgery, CT other than carboplatin, RT of the RP, psychiatric disorder, or language limitation	The Ipsilateral ureter represented the caudal and lateral boundary of resection; the renal artery was described as the cranial and the crus of the diaphragm as the posterior resection boundary.	None
**Ladi-Seyedian et al, ( 25 ) 2023**	Retrospective, single-arm, multicenter (Conference abstract)	1-5 cm, ipsilateral, synchronous or metachronous	NA	NA	NA	NA
**Matulewicz et al. ( 26 ), 2024**	Retrospective, single-arm, single-center	CSII or relapsed CSI seminoma isolated in RP	Normal AFP	Previous CT and elevated AFP	Bilateral full-template and pelvic lymph node dissection for equivocal or enlarged pelvic adenopathy	According to final pathology
**Thor et al. ( 27 ), 2023**	Prospective, single-arm, multicenter (Conference abstract)	CS IIA or IIB	NA	NA	NA	NA
**Warszawski et al. ( 28 ), 1997**	Retrospective, double-arm, single-center	CS I or II§	NA	NA	Excision of para-aortic and para-caval lymph nodes from renal pedicle to aortic bifurcation	NA

AFP: Alpha Fetoprotein; ß-hCG: Beta Human Chorionic Gonadotropin; CS: Clinical Stage; CT: Chemotherapy; LDH: Lactate Dehydrogenase; NA: Not Available; RP: Retroperitoneal; RT: Radiation Therapy; ULN: Upper Limit of Normal. / *Until the enrollment of 31 patients, the eligibility criteria were lymph nodes up to 2 cm. / §From all the patients included in the study, we only considered for our analysis those who were staged as CS IIA or IIB. / ^+^The authors reported that only one patient received a single cycle of carboplatin for pN2 disease (lymph node 5 cm and extra-nodal extension).

The seven studies included 331 male patients diagnosed with CS IIA/B pure testicular seminomas and underwent RPLND in centers across North America, Canada, and Europe. Among the studies providing detailed information about the CS of the patients at the time of RPLND, 110 (33.2%) were classified as CS IIA, and 51 (15.4%) patients were CS IIB. In one study, 16 patients (35%) received adjuvant treatment in addition to surgery based on pathological findings. Thus, for the pooled analysis of the RFS and recurrence rate, we only considered the data of the patients who underwent RPLND followed by surveillance ( 26 ). Additionally, three studies reported upstaging rates ranging from 30–44% ( 22 , 25 , 26 ).

The median age of patients was 37 years (range: 34–42.6 years), and the follow-up ranged from 17–79 months. The median size of the clinical lymph nodes was 1.86 cm (range: 1.6–2.3 cm), with one study restricting their inclusion criteria to patients with retroperitoneal adenopathy measuring up to 3 cm ( 22 ). Despite variations in surgical management templates across the studies, the prevailing approach involved modified ipsilateral RPLND, employing either an open or robotic surgical approach, while the decision to adopt a bilateral template rested at the surgeon’s discretion. A bilateral full-template was performed routinely in one study, with pelvic lymph node dissection for patients with enlarged pelvic lymph nodes ( 26 ). The clinical and surgical baseline characteristics of the included patients are presented in Table-2 .

**Table 2:** Individual characteristics of the included studies.

Study	Patients, n	Age,^*^ years	Follow-up,^*^ months	Clinical Stage		N-S RPLND, n (%)	Lymph Node Size,^*^ cm	Surgical approach		Positive Lymph Node,* n	Diameter Lymph Node,* cm	Pathologic Nodal Stage			Upstaging,n (%)
				IIA, n (%)	IIB, n (%)			Open, n	Robotic, n			pIIA, n (%)	pIIB, n (%)	N0, n (%)	
**Daneshmand et al. ( 22 ), 2023**	55	34 (20–64)✝	33	44 (80)	11 (20)	48 (87)	1.6 (1.0–3.5)✝	NA	NA	1 (0–12)✝	2.30 (0.03–12.3) ✝	21 (38)	31 (56)	9 (16)	24 (44)
**Heidenreich et al. ( 23 ), 2023**	30	39.1 (34–52)§	21.5	NA	NA	30 (100)	2.3 (1.3–4.5)§	27	3	1 (1–2)§	2.4 (1.3–4.5)§	19 (63)	11 (37)	3 (10)	NA
**Hiester et al. ( 24 ), 2023**	33	37 (30–42)§	32	13 (39)	20 (61)	33 (100)	2.0 (1.4–2.5)§	14	19	1 (1–4)§	2.8 (2.0–3.7)§	NA	NA	3 (9)	NA
**Ladi-Seyedian et al, ( 25 ) 2023**	94	35 (31–43)§	NA	NA	NA	87 (93)	NA	92	2	NA	NA	28 (30)	53 (56)	10 (11)	28 (30)
**Matulewicz et al. ( 26 ), 2024**	45	36 (32–43)§ (22–66)✝	18.5	NA	NA	32 (71)	1.8 (1.4–2.2)§	NA	NA	2 (1–3)§	2.0 (1.4–2.5)§	10 (22)	30 (67)	2 (4.4)	20 (44)
**Thor et al. (27), 2023**	61	42.6 (25–79)✝	17	46 (75)	14 (23)	NA	NA	37	24	NA	NA	NA	NA	NA	NA
**Warszawski et al. ( 28 ), 1997**	13	NA	79	7 (54)	6 (46)	NA	NA	13	0	NA	NA	NA	NA	NA	NA

CS: Clinical Stage; NA: Not Available; N-S RPLND: Nerve Sparing Primary Retroperitoneal Lymph Node Dissection; Pathologic Nodal Stage: pIIA, pIIB, N0.

*Mean or Median

§(IQR – Interquartile range)

+(Range)

### Overall analysis

**Figure 2:**
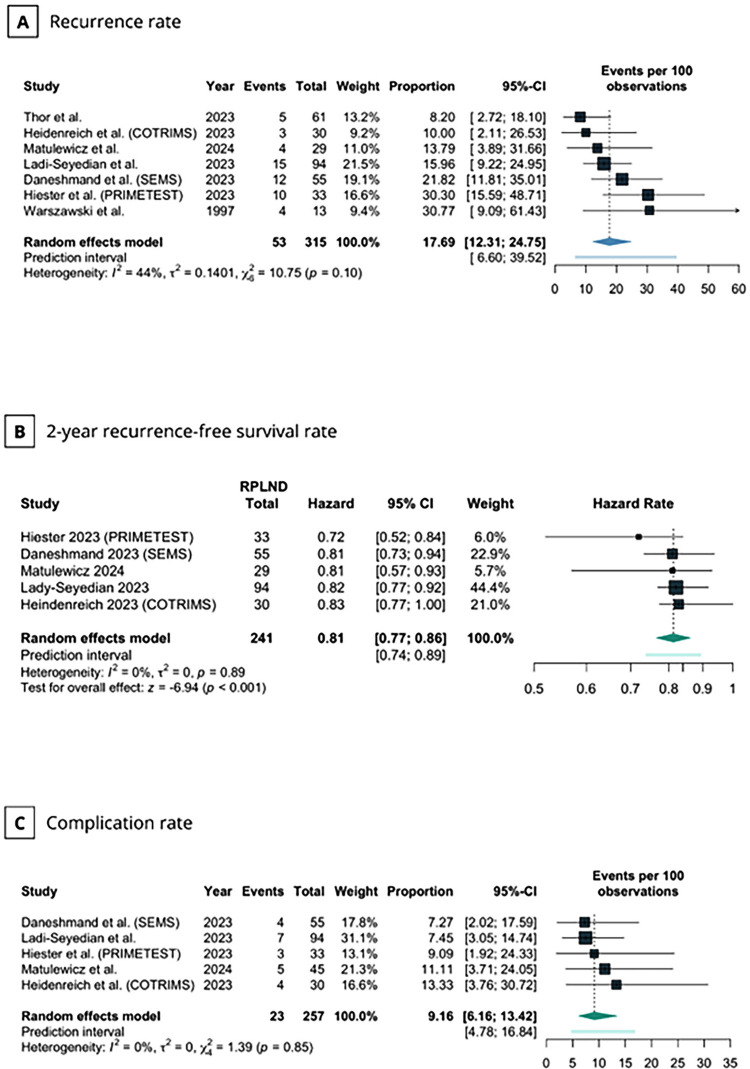
- Meta-analysis of primary endpoints after primary RPLND in patients with CS IIA/B testicular seminoma.

Recurrence rates across studies ranged from 8.2–30.8%. In the pooled analysis, the recurrence rate was 17.69% (95% CI 12.31–24.75; I^2^=44%; Figure-2A ). Four studies reported a 2-year RFS rate, while in one study, it was derived from IPD reconstructed from the KM curve, which ranged from 72–83% ( 25 ). The pooled 2-year RFS was 81% (95% CI 0.77–0.86; I^2^=0%; Figure-2B ).

(A) Recurrence rate; (B) 2-year recurrence-free survival rate; and (C) Complication rate.

CI, confidence interval; COTRIMS, Cologne Trial of Retroperitoneal Lymphadenectomy in Metastatic Seminoma; PRIMETEST, Primary Retroperitoneal Lymph Node Dissection in Patients with Seminomatous Testicular Germ Cell Tumors with Clinical Stage IIA/B trial; RPLND, Retroperitoneal lymph node dissection; SEMS, Surgery in Early Metastatic Seminoma trial.

**Figure 3:**
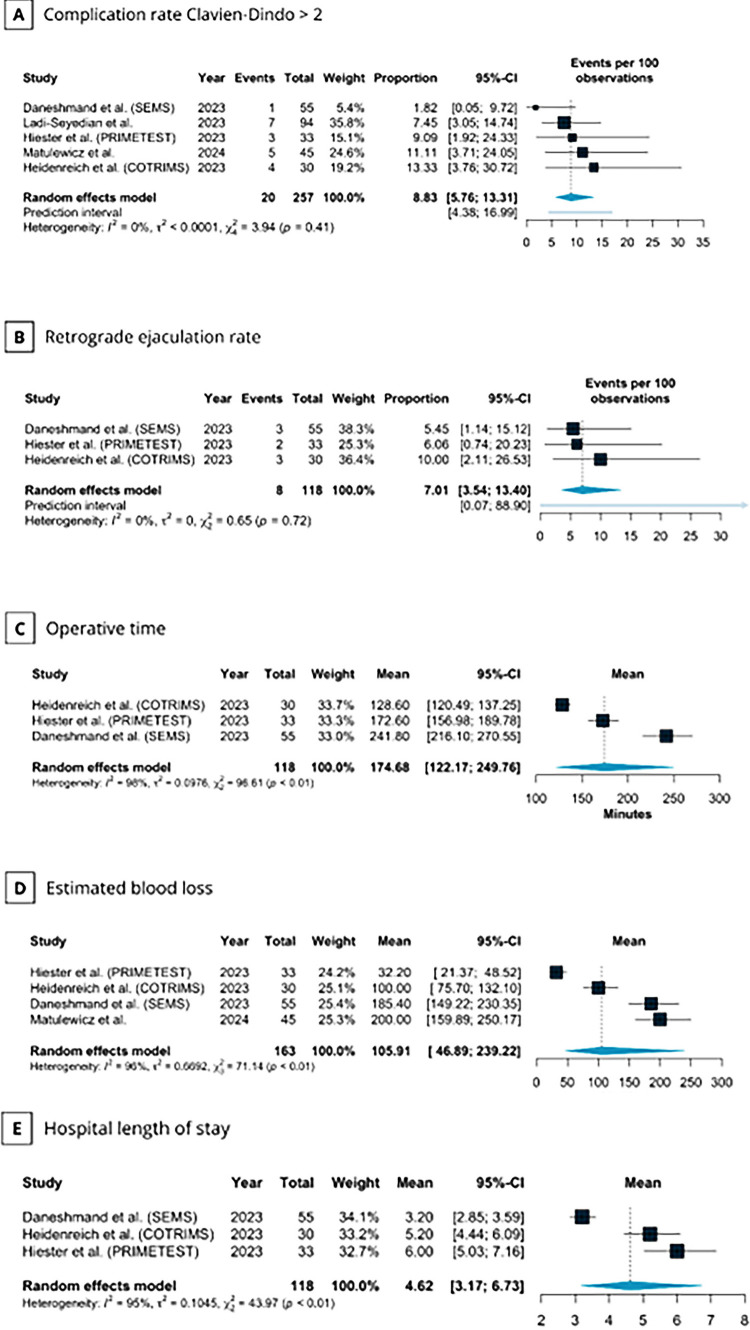
Meta-analysis of secondary endpoints after primary RPLND in patients with CS IIA/B testicular seminoma.

Five studies reported a total of 23 complications after RPLND, with rates ranging from 7.27–13.33%. In the pooled analysis, RPLND was associated with a complication rate of 9.16% (95% CI 6.16–13.42; I^2^=0%; Figure-2C ). Of these, 20 were classified as CD grade > 2. The pooled complication rate for CD grade > 2 was 8.83% (95% CI 5.76–13.31; I^2^=0%; Figure-3A ).

(A) Complication rate Clavien-Dindo > 2; (B) Retrograde ejaculation rate; (C) Operative time (min); (D) Estimated blood loss (mL); and (E) Hospital length of stay (days).

CI, confidence interval; COTRIMS, Cologne Trial of Retroperitoneal Lymphadenectomy in Metastatic Seminoma; PRIMETEST, Primary Retroperitoneal Lymph Node Dissection in Patients with Seminomatous Testicular Germ Cell Tumors with Clinical Stage IIA/B trial; RPLND, Retroperitoneal lymph node dissection; SEMS, Surgery in Early Metastatic Seminoma trial

The nerve-sparing procedure ranged from 71–100%, and three studies reported retrograde ejaculation rates ranging from 5.45–10%. The pooled retrograde ejaculation rate was 7.01% (95% CI 3.54–13.40; I^2^=0%; Figure-3B ).

Among the included trials, the mean operative time was 174.68 minutes (95% CI 122.17–249.76 minutes; I^2^=98%; Figure-3C), the mean blood loss was 105.91 mL (95% CI 46.89–239.22 mL; I^2^=96%; Figure-3D), and the median hospital stay was 4.62 days (95% CI 3.17–6.73 days; I^2^=95%; Figure-3E ).

### Sensitivity analysis

The recurrence rate was the only primary outcome presenting elevated between-study heterogeneity (I^2^=44%). Consequently, a subgroup analysis was conducted by pooling the data exclusively from prospective trials, revealing a recurrence rate of 16.76% (95% CI, 8.80–29.58; Supplementary Figure-1A APPENDIX ). Nevertheless, the observed heterogeneity remained elevated (I^2^=66%). Leave-one-out sensitivity analysis for the 2-year RFS rate revealed that no single study significantly influenced the heterogeneity or the overall pooled result ( Supplementary Figure-1B ).

### Quality assessment

Supplementary Figure-2 APPENDIX summarizes the individual risk of bias assessments of studies performed according to the Cochrane Collaboration’s tool ROBINS-I. Four studies were rated as “low risk” of bias, and three were “moderate risk” due to their potential to introduce confounding factors and bias in patient selection. Moreover, their retrospective design may influence the determination of patient exclusion criteria based on specific findings such as outcomes, comorbidities, laboratory results, and treatment history ( 25 , 26 , 28 ).

## DISCUSSION

In this systematic review and single-arm meta-analysis comprising seven studies and 331 non-overlapping patients, we comprehensively evaluated RPLND as a first-line therapy for CS IIA/B seminomas. Our main findings were that the recurrence rate was 17.69% (95% CI 12.31–24.75), the 2-year RFS was 81% (95% CI 0.77–0.86), and the complication rate was 9.16% (95% CI 6.16–13.42).

Currently, para-aortic and pelvic radiation therapy or systemic chemotherapy is the standard treatment option for CS IIA/B seminomas, resulting in high rates of cancer-specific survival (> 97%) and low relapse rates, ranging from 9–24% in these tumors ( 29 , 30 ). While chemotherapy (either with three cycles of bleomycin, etoposide, and cisplatin or four cycles of etoposide and cisplatin) is the preferred regimen in CS IIC, in CS IIA/B, both treatment modalities seem to be equally effective. Direct comparative studies between chemotherapy and radiation therapy are scarce and primarily confined to retrospective analyses. Although none of these studies have demonstrated significant differences in survival rates, a noticeable trend in relapse was observed in patients diagnosed with CS IIB who underwent radiation therapy ( 30 – 32 ).

However, both treatments are associated with immediate and long-term side effects, including cardiovascular disease, metabolic disorders, endocrine disorders, hypogonadism, infertility, and secondary hematological or solid tumors ( 8 , 33 ). Therefore, the primary goal in managing patients with testicular GCTs is to minimize the long-term toxicity associated with treatment while preserving therapeutic efficacy. Studies have explored de-escalation strategies, such as reducing radiation fields, combining radiation therapy with one cycle of carboplatin, and de-escalating systemic chemotherapy regimens (34–36). Furthermore, earlier database reviews reported a 5-year overall survival of 92% in patients who underwent primary RPLND in this setting, underscoring this surgery as a feasible therapeutic option ( 37 ).

The recurrence rate of primary RPLND for CS IIA/B seminomas was 17.69% (95% CI 12.31–24.75), while the 2-year RFS was 81% (95% CI 0.77–0.86). When compared with the standard recommended treatments, the reported relapse rates of radiation therapy ranged from 9–24%, while the 5-year RFS in CS IIA and IIB are 92% and 90%, respectively ( 38 , 39 ). Although a few studies have reported outcomes on chemotherapy regimens for CS IIA/B seminomas, the recurrence rates were 0%, and the 5-year RFS was 100% ( 40 , 41 ).

The elevated RFS rate indicates that these patients who underwent RPLND were free from adverse events associated with chemotherapy or radiation therapy at a 2-year follow-up. Furthermore, even those who experienced recurrence were still successfully treated with standard therapies. For example, in the SEMS trial, among 12 patients who experienced a recurrence, ten were treated with chemotherapy, and two underwent additional surgery ( 22 ). In the COTRIMS trial, three patients (10%) developed an outfield relapse at 4, 6, and 9 months postoperatively and were salvaged by systemic chemotherapy ( 23 ). In the PRIMETEST trial, the median time to relapse was 6 months, and all these patients were successfully treated with systemic chemotherapy ( 24 ). Additionally, Matulewicz et al. reported four relapses in the surveillance group after RPLND, all of which were treated with chemotherapy, with no retroperitoneal relapses observed ( 26 ).

Importantly, the RPLND template differed across the studies. Most patients underwent modified ipsilateral RPLND ( Table-1 ), based on mapping studies of retroperitoneal metastasis, aiming to limit the extent of dissection in anatomic regions thought to be at a decreased risk of metastatic dissemination and to avoid ejaculatory dysfunction. The RPLND template is directly related to testis lymphatic drainage. The right-sided testicular drainage included the interaortocaval lymph nodes, followed by the precaval and paracaval nodes, whereas left-sided drainage included the left para-aortic and preaortic lymph nodes ( 42 ). The retroperitoneal dissemination contralateral to the testis compromised by the tumor is more common with right-sided tumors than in left-sided tumors and is usually associated with large-volume disease ( 43 ). The standard bilateral RPLND template limits are the ureters (lateral), bifurcation of iliac vessels (inferior) and renal hilum (superior). Recent data suggests that modified ipsilateral RPLND might underestimate the risk of contralateral retroperitoneal metastases in almost 32% of the patients ( 44 ).

In CS I, the main risk factors for relapse are testicular tumor size and stromal invasion of the rete testis ( 45 ). The risk of relapse in unselected CS I patients varies between 12–20% at five years, with 17% in the largest series ( 46 ). The absence of both factors indicates a low risk of recurrence of around 6% ( 47 ). Recurrences, when present, occur mainly in the retroperitoneum during the first two years ( 48 ). Among the studies included in this meta-analysis, only one performed a bilateral RPLND and had no retroperitoneal recurrence ( 26 ). Therefore, considering the characteristics of the primary tumor, improving risk stratification and performing subgroup analyses could better individualize the RPLND template that needs to be performed.

The rate of patients with no evidence of regional lymph node involvement (N0) ranged from 0–16%. This range is consistent with rates observed in RPLND for non-seminoma tumors, where surgery has been the standard treatment modality for numerous years ( 44 ). The definitive pathology is a benefit exclusively attainable through surgery. Consequently, over the past several decades, patients with N0 status may have been subjected to overtreatment with primary radiation therapy or chemotherapy, potentially leading to unnecessary acute and long-term toxicity and requiring long-term follow-up. This situation underscores the necessity for refinement in pre-imaging techniques and the development and routine use of molecular serum markers. For example, measuring miR371, the most promising biomarker, levels could aid in distinguishing metastatic and non-metastatic diseases, thereby preventing unnecessary treatments ( 49 ).

Conversely, surgical interventions are susceptible to perioperative complications. Our study revealed an overall complication rate of 9.16%, with 8.83% classified as CD grade > 2, exhibiting no heterogeneity across the various studies. Heister et al. observed one patient with a post-operative ileus that required revision surgery, two with pulmonary embolisms and one with lymphocele requiring drainage ( 24 ). Matulewicz et al. reported two patients with chylous ascites requiring bedside paracentesis, two with infections that resolved with oral antibiotics, and one with wound breakdown resolved with conservative treatment ( 26 ). Daneshmand et al. described four patients with complications: one with incision ulceration, one with ileus, one with ileus and pulmonary embolism and one with chylos ascites ( 22 ). Ladi-Seyedian et al. observed seven (7.5%) complications, including ileus, incision ulceration, pulmonary embolism and chylous ascites ( 25 ). Heidenreich et al. reported four complications: two retroperitoneal lymphoceles, one ileus and one chylos ascites, but did not provide the resolution of these complications ( 23 ). In contrast, Thor et al. did not specify the complications encountered by seven patients ( 27 ).

It is well-established that post-chemotherapy RPLND is a more challenging procedure attributed to desmoplastic reaction, with complication rates reaching 24.7% ( 50 ). Early initiation of surgery during the management of CS II seminomas may mitigate the morbidity linked to RPLND in patients with residual masses following chemotherapy. Indeed, evidence supports the safety of this approach when performed using minimally invasive techniques ( 51 ). Moreover, a limited number of long-term complications of RPLND were reported in the studies, including issues such as incisional hernia, ejaculatory dysfunction, ureteral obstruction, or intestinal obstruction. Notably, these low complication rates observed are associated with an increased number of procedures performed in those centers. This result highlights the importance and preference of such RPLND-specialized centers to ensure treatment efficacy and patient safety.

Nerve-sparing surgery was performed on most patients in the studies, with rates ranging from 71–100%. It should be pointed out that a 7% retrograde ejaculation rate was observed, which may affect men’s pleasure. Furthermore, infertility has been recognized as a significant concern for survivors of testicular cancer, especially considering its elevated incidence among young men ( 52 ). Both hypogonadism and infertility stand as potential adverse events associated with radiation therapy and chemotherapy ( 8 , 33 ). In this context, nerve-sparing primary RPLND holds an advantage by providing definitive staging with low rates of ejaculatory disorders ( 53 ). However, it has been associated with higher rates of in-field recurrence and complications. Therefore, it should be performed by experienced surgeons ( 54 ).

The included studies’ main limitations are the limited follow-up and long-term oncologic data, which are essential for consolidating progression-free survival rates. Additionally, the surgical template and inclusion criteria varied among the studies. Given the well-established association between disease volume and an increased recurrence rate, these variations may explain the elevated between-study heterogeneity observed in this outcome. However, consistent results were found in the leave-one-out sensitivity analysis. Finally, there is a paucity of prospective randomized controlled trials comparing upfront RPLND with radiation therapy or chemotherapy.

Although a recently published meta-analysis has already evaluated RPLND for CS II seminomas, our study has some key advantages. First, we included three additional studies, adding 200 patients not considered in the prior meta-analysis (25–27). Second, Parizi et al. included two studies with overlapping populations, double-counting individuals in the evidence synthesis ( 22 , 55 , 56 , 57 ). Third, we pooled the 2-year RFS rates estimated from the published KM curves. Fourth, additional endpoints, such as operative time, estimated blood loss, and hospital stay length, were analyzed. Finally, we also performed a sensitivity analysis, including a leave-one-out analysis, to ensure the robustness of our findings.

To the best of our knowledge, this study analyzed the most recent RPLND data in this context. It imparts pertinent information concerning recurrence rates and complications, illustrating the proposed procedure’s safety and advantages as the primary approach for treating CS IIA/B seminomas.

## CONCLUSIONS

This single-arm meta-analysis of studies that evaluated patients who underwent RPLND to treat CS IIA/IIB seminomas demonstrated favorable RFS rates with low recurrence rates and complications. Additional studies are warranted to assess the comparative effectiveness of RPLND versus alternative strategies for treating this patient cohort and to investigate long-term and post-recurrence outcomes following RPLND.

## APPENDIX

Supplementary Figure 1A - Recurrence rate including only prospective trials.


