# Supplementary material for: Primary Retroperitoneal Lymph Node Dissection for Clinical Stage II A/B Seminomas: A Systematic Review and Meta-Analysis
Source: Int Braz J Urol. 2024 Apr 25;50(4):415–32. doi: 10.1590/S1677-5538.IBJU.2024.0134 (PMC11262717; doi:10.1590/S1677-5538.IBJU.2024.0134)

APPENDIX

Supplementary Figure 1A - Recurrence rate including only prospective trials.

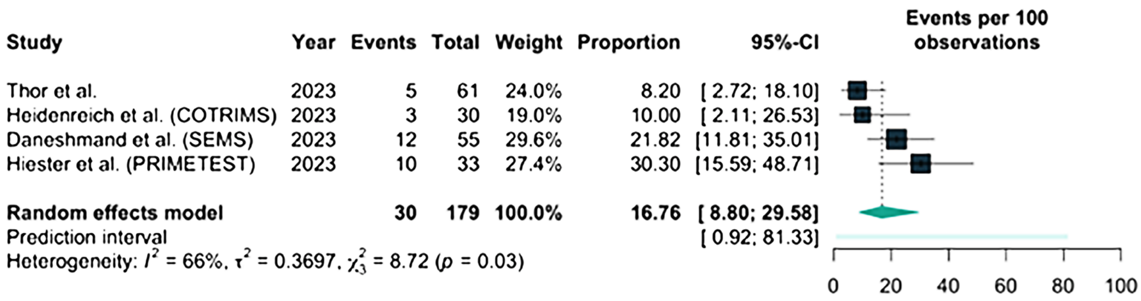

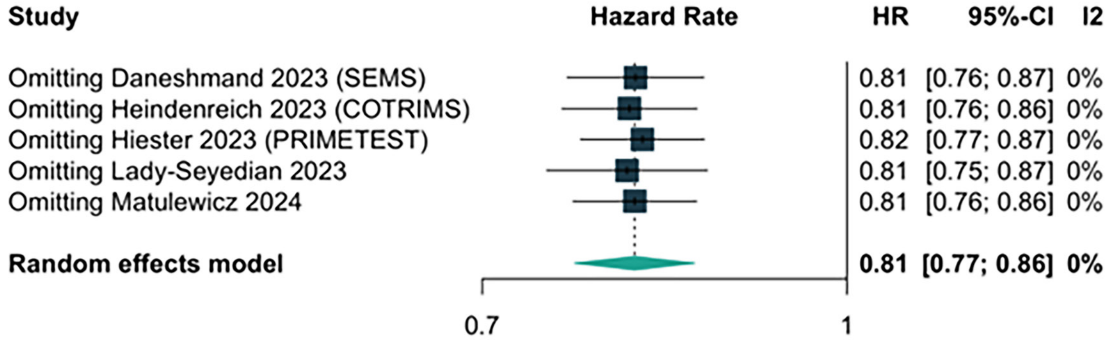

Supplementary Figure 2 - Summary of Risk of Bias assessment using ROBINS-I tool.

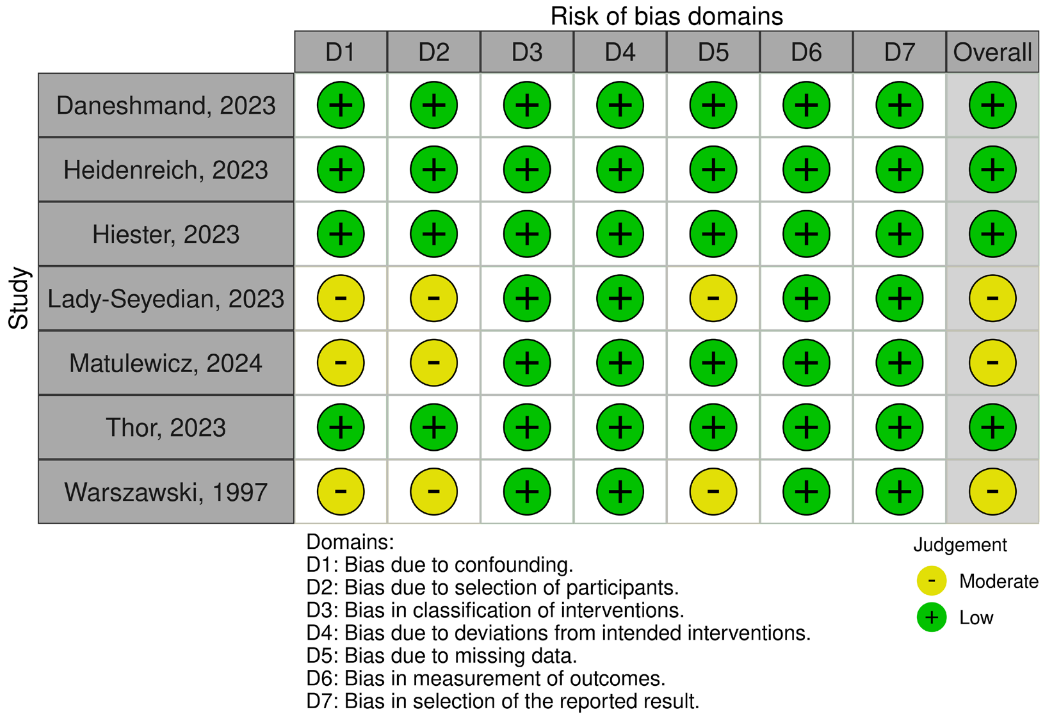

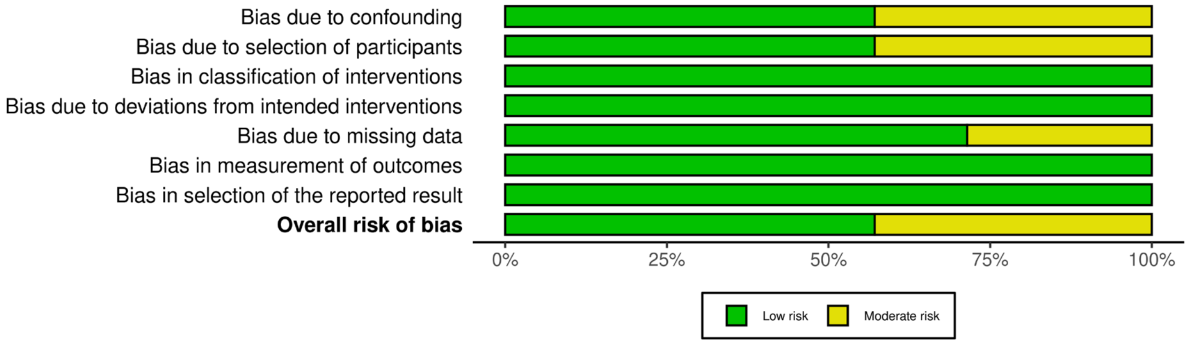

Supplement: Supplementary file 1 [file 1677-6119-ibju-50-04-0415-suppl01.pdf]
